# Our health counts: population-based measures of urban Inuit health determinants, health status, and health care access

**DOI:** 10.17269/s41997-018-0111-0

**Published:** 2018-10-09

**Authors:** Janet Smylie, Michelle Firestone, Michael W. Spiller, Tungasuvvingat Inuit

**Affiliations:** 1 Well Living House and Centre for Urban Health Solutions, Li Ka Shing Knowledge Institute, St. Michael’s Hospital, 30 Bond Street, Toronto, ON M5B 1W8 Canada; 2 Department of Family and Community Medicine, St. Michael’s Hospital, 30 Bond Street, Toronto, ON Canada; 3 Dalla Lana School of Public Health, University of Toronto, Toronto, Canada; 4 Department of Sociology, Cornell University, 323 Uris Hall, Ithaca, New York, NY 14853 USA; 5 Ottawa, ON Canada

**Keywords:** Urban Inuit, Respondent-driven sampling, Indigenous health, Population health assessment, Community-partnered research, Data linkage, Inuits urbains, Échantillonnage en fonction des répondants, Santé autochtone, Évaluation de la santé de populations, Recherche en partenariat avec le milieu associatif, Couplage de données

## Abstract

**Objective:**

Health determinants and outcomes are not well described for the growing population of Inuit living in southern urban areas of Canada despite known and striking health disparities for Inuit living in the north. The objective of this study was to work in partnership with Tungasuvvingat Inuit (TI) to develop population prevalence estimates for key indicators of health, including health determinants, health status outcomes, and health services access for Inuit in Ottawa, Canada.

**Methods:**

We employed community-based respondent driven sampling (RDS) and a comprehensive health assessment survey to collect primary data regarding health determinants, status, and service access. We then linked with datasets held by the Institute for Clinical Evaluative Sciences (ICES), including hospitalization, emergency room, and health screening records. Adjusted population-based prevalence estimates and rates were calculated using custom RDS software.

**Results:**

We recruited 341 Inuit adults living in Ottawa. The number of Inuit living, working or accessing health and social services in the City of Ottawa was estimated to be 3361 (95% CI 2309–4959). This population experiences high rates of poverty, unemployment, household crowding, and food insecurity. Prevalence of hypertension (25%; 95% CI 18.1–33.9), chronic obstructive pulmonary disease (6.7%; 95% CI 3.1–10.6), cancer (6.8%; 95% CI 2.7–11.9), and rates of emergency room access were elevated for Inuit in Ottawa compared to the general population. Access to health services was rated fair or poor by 43%. Multiple barriers to health care access were identified.

**Conclusions:**

Urban Inuit experience a heavy burden of adverse health determinants and poor health status outcomes. According to urban Inuit in Ottawa, health services available to Inuit at the time of the study were inadequate.

## Résumé

**Objectif:**

Les déterminants de la santé et leurs résultats dans la population croissante d’Inuits vivant dans les agglomérations urbaines du Sud du Canada ne sont pas bien décrits malgré les disparités de santé connues et évidentes entre les Inuits vivant dans le Nord et le reste de la population. Notre étude visait à produire, en partenariat avec l’organisme Tungasuvvingat Inuit (TI), des estimations sur la prévalence d’indicateurs de santé clés dans la population, notamment les déterminants de la santé, les résultats sanitaires et l’accès aux services de santé pour les Inuits vivant à Ottawa, au Canada.

**Méthode:**

Nous avons eu recours à l’échantillonnage en fonction des répondants (EFR) en milieu ouvert et à un questionnaire global d’évaluation de la santé pour recueillir des données primaires sur les déterminants de la santé, l’état de santé et l’accès aux services. Nous avons ensuite couplé ces données avec celles des fichiers de l’Institut de recherche en services de santé, notamment sur les hospitalisations, les visites aux urgences et le dépistage. Des estimations et des taux de prévalence dans la population ajustés ont été calculés à l’aide d’un logiciel d’EFR conçu sur mesure.

**Résultats:**

Nous avons recruté 341 Inuits d’âge adulte vivant à Ottawa. Le nombre d’Inuits vivant, travaillant ou utilisant les services sociaux et de santé dans la ville d’Ottawa a été estimé à 3 361 (IC de 95 % : 2309-4959). Les taux de pauvreté, de chômage, de logements surpeuplés et d’insécurité alimentaire sont très élevés dans cette population. La prévalence de l’hypertension artérielle (25 %; IC de 95 % : 18,1-33,9), de la maladie pulmonaire obstructive chronique (6,7 %; IC de 95 % : 3,1-10,6) et du cancer (6,8 %; IC de 95 % : 2,7-11,9) et les taux de visites aux urgences étaient élevés chez les Inuits à Ottawa comparativement à la population générale. L’accès aux services de santé a été jugé moyen ou mauvais par 43 % des répondants. De nombreuses barrières à l’accès aux soins de santé ont été mentionnées.

**Conclusions:**

Des déterminants de santé défavorables et de mauvais résultats sanitaires imposent un lourd fardeau aux Inuits urbains. De l’avis des intéressés, les services de santé dont disposaient les Inuits vivant à Ottawa au moment de l’étude étaient insuffisants.

## Introduction

The striking health inequities experienced by the Inuit living in northern Canada are described in a growing body of literature (Tait 2008; Oliver et al. 2012; Wilkins et al. 2008; Peters 2012). Despite Canada’s global status as a relatively affluent country, many health outcomes for Inuit are comparable to health outcomes in low-income countries (Tait 2008; Oliver et al. 2012; Wilkins et al. 2008; Kelly et al. 2008). For example, rates of infant mortality in Inuit inhabited areas in northern Canada are 3.6 times higher compared to non-Inuit inhabited areas (Wilkins et al. 2008), and rates of tuberculosis are 20 times higher in Nunavut than for the general Canadian population (Nguyen et al. 2003; Public Health Agency of Canada 2015; Macdonald et al. 2011). The gap in life expectancy between Inuit and other Canadians is growing. Currently, life expectancy at birth in Inuit inhabited areas is 10–17 years less than that of the general Canadian population. Life expectancy estimates in Inuit inhabited areas has remained stable or even decreased slightly between 1991 and 2001, during which time life expectancy improved for the general Canadian population (Wilkins et al. 2008; Statistics Canada 2008). Between 33% and 37% of Inuit children and 44% of Inuit adults have one or more chronic health conditions (Tait 2008; Guèvremont and Kohen 2013). This growing burden of disease has been linked to marked disparities in the social determinants of health, including access to medical services (Garner et al. 2010; National Collaborating Centre for Aboriginal Health 2009). These disparities in turn have roots in the preceding cultural, socio-economic, and political transitions accompanying European colonization, an experience that is shared by Inuit populations internationally across circumpolar regions (Peters 2012; Inuit Tapiriit Kanatami 2007).

Urban Inuit populations are growing rapidly (Statistics Canada 2008; Gionet 2008). Inuit travel to southern urban centres for health, social services, work, school, and other opportunities not available in the north (Patrick and Tomiak 2008). According to the 2011 National Household Survey, 27% of Inuit in Canada lived outside of Inuit Nunangat.^1^ Of this group, four out of ten lived in large urban centres with populations of greater than 100,000 persons (Statistics Canada 2011). Ottawa-Gatineau has the largest urban Inuit population outside Inuit Nunangat, followed by Edmonton, Montreal, and Yellowknife (Statistics Canada 2017).

Tungasuvvingat Inuit (TI) is a provincial service provider whose mission is to meet the rapidly growing, complex and evolving needs of Inuit in Ontario by providing interconnected and holistic programs that contribute to the health and well-being of Inuit across the lifecycle. TI offers a diverse range of services to the Inuit community in Ottawa and across Ontario, including health promotion, housing, employment, and trauma and addiction programs. In 2014, Akausivik Inuit Family Health Team (AIFHT) was formed to respond more specifically to the medical needs of Inuit in Ottawa. AIFHT provides culturally appropriate, interdisciplinary primary care to the Inuit.

Systemic deficiencies in the identification of Indigenous people in Canada’s health information systems preclude quality demographic and population-based health assessment for urban Inuit, which in turn impedes responsive service planning and implementation. The main problem is that core health data sources in Canada do not have an Inuit identifier. Upon release of the 2006 census findings, TI identified that census estimates of the number of Inuit living in Ottawa (*N* = 725) represented a significant underestimate based on their roster of Inuit with Ottawa addresses.

In 2011, challenges with respect to enumeration of Inuit living outside of the Inuit Nunangat were compounded when Canada substituted the long-form census with a National Household Survey. Population-based health information for Inuit living in northern Canada have been obtained using geocoded linkage to areas where a substantial proportion of the population is Inuit and surveys drawing on the northern Inuit samples in the 2006 and prior censuses (Tait 2008; Oliver et al. 2012; Wilkins et al. 2008; Guèvremont and Kohen 2013). Both of these methods do not include urban Inuit.

The purpose of this study was to fill gaps in urban Inuit health information by working in partnership with TI. Specifically, we aimed to better understand the population health service needs for Inuit living, working or accessing health and social services in the City of Ottawa by developing population-based prevalence estimates for key indicators of health, across health determinants, health status outcomes, and health services access for this population. This study of Inuit adults is part of a larger three-site urban Indigenous health database development research project entitled Our Health Counts (Smylie et al. 2011; Firestone et al. 2014a), which included adult and child database development for urban First Nations, Inuit, and Métis communities in Ontario.

## Methods

### Community-based participatory research partnership

In keeping with current ethical and scientific standards in Indigenous health research (Canadian Institutes of Health Research, Natural Sciences and Engineering Research Council of Canada, Social Sciences and Humanities Research Council of Canada 2014; Canadian Institutes of Health Research 2007), the academic research team engaged in a community-based participatory research partnership with the TI to complete this study. This approach built on previously successful research partnerships and involved active participation of the TI on the research team at all stages of the research from design to dissemination (Smylie et al. 2006; Smylie et al. 2009). Community-based Inuit staff of TI implemented recruitment and survey administration. TI led the governance and management of the data. Inuit community research team members strove to ensure that all study materials and protocols met the highest standards of local Inuit community relevance (Smylie et al. 2012). Formal ethics approval was provided by the Research Ethics Board of Saint Michael’s Hospital.

### Respondent-driven sampling

In the absence of an existing reliable population-based sampling frame for Inuit in Ottawa, we used respondent-driven sampling methods (RDS). RDS is an innovative chain-referral sampling approach that is increasingly being used to recruit samples from “hidden” populations. RDS methods draw on Markov theory and social network information to produce population-based estimates samples collected via social networks (Johnston and Sabin 2010; Heckathorn et al. 2002).

For sample size calculation, we used 95% confidence intervals (CI) and survey item prevalence ranges from 10% to 75%. Due to its complexity, RDS requires a larger sample size than simple random samples to generate population estimates of similar variance. We assumed a RDS to simple random sample size ratio (also referred to as the design effect) of 2 (Salganik 2006). Based on this formula, we originally aimed to recruit 500 Inuit adults; however, this target was refined based on recruitment patterns and length of RDS chains as the study progressed.

Inclusion criteria for the adult study were self-identification as Inuk, ≥ 18 years old or younger than 18 years and parenting a child or children, residency in the City of Ottawa and/or employment in the City of Ottawa, and/or recipient of health and/or social services in the City of Ottawa.

We initiated RDS sampling by selecting eight “seeds” who represented a diverse demographic of Inuit people living in Ottawa with respect to gender, age, family size, occupation, and where they lived (Johnston and Sabin 2010). Each seed was given three coupons and asked to refer a friend, acquaintance, or family member to the study. Subsequent participants were given three to five recruitment coupons. In order to increase the number of completed surveys and diversify the sample, three additional seeds were added six weeks into the study. Participants received a monetary compensation for their time and participation, which consisted of $20 for completion of the survey and an additional $10 for every person recruited into the study.

Following standard RDS procedures, we collected social network information from each study participant at the time of recruitment. Specifically, coupons were uniquely numbered so we could determine who recruited whom and we asked each respondent how many Inuit they knew who were living, working, or accessing health and social services in the city of Ottawa as well as what their relationship was to the person who recruited them (i.e., relative; girlfriend/boyfriend/partner or spouse; friend; acquaintance; stranger).

Recruitment was initiated in May 2010 and lasted 6 months. Overall, RDS sampling was extremely successful. After a growth period consistent with RDS, recruitment was extremely brisk. We issued 1374 coupons, and 330 of these were redeemed. The decision was made to stop recruitment in October 2010 as recruitment had tapered, and the sample of Inuit adults (*N*=341) was deemed adequate based on the estimated sampling variability for important study estimates.

Assumptions regarding recruiter-recruit relationships, minimum recruitment chain length, sampling with replacement, and network segregation were met (Gile and Handcock 2010). The longest recruitment chain was 11 waves, much longer than the 6–7 waves typically deemed sufficient in RDS to overcome bias due to the non-random selection of seeds (Wejnert 2010). Based on these facts, our team was confident that our sample would provide rigorous population-based prevalence estimates. The estimated design effect for the select variables of hypertension, ER access, hospitalization, and personal income were 2.88, 3.40, 3.44, and 3.66 respectively.

### Sources of data

Following recruitment and consent, participants completed a community-tailored and comprehensive Inuit-specific health assessment survey in either Inuktitut or English, administered in person by a trained, bilingual Inuit community research team member who used computer-assisted personal interviewing. To ensure content validity, this Inuit Respectful Health Assessment Survey tool was constructed according to seven interconnected priority health assessment domains identified through community-based concept mapping (Firestone et al. 2014b). For each of these domains, the academic and Inuit research team identified relevant survey questions in the existing literature (Statistics Canada 2012; First Nations Information Governance Committee (FNIGC) 2008; Borenstein et al. 2013) or in cases where relevant questions did not exist, developed new questions. We piloted the survey with urban Inuit not eligible to participate in the study and subsequently made minor modifications to improve face validity. Using IBM® SPSS® Data Collection Author (IBM, SPSS 2000), we then developed the computerized version of the survey. The full questionnaire is available at http://tungasuvvingatinuit.ca/wp-content/uploads/2018/05/Appendix-D-E-Adult-and-Child-Survey.pdf

Rates of overcrowding were calculated using the Statistics Canada definition of overcrowding (Statistics Canada 2010). We inquired about food insecurity by asking “Were there times when the food for you and your family just did not last (and there was no money to buy more)?” Self-reported health status measures included chronic health conditions as diagnosed by a health care provider (“Have you been told by a health care provider that you have any of the following health conditions?”): injury, upper and lower respiratory tract infection, and severe pain over the past 12 months. Access to care measures included self-rated access to care (“Overall, how would you rate the availability of health services in your community?”) and whether or not the respondent had experienced one or more items on a list of barriers to accessing health care, including a specific list of communication/cross-cultural barriers.

Upon completion of recruitment and survey, with consent, our sample of Inuit adults was linked to data holdings at the Institute of Clinical Evaluative Sciences (ICES), including provincial records of emergency room and hospital visits (Canadian Institute for Health Information – Discharge Abstract Database and National Ambulatory Care Reporting System: Emergency), participation in preventive health screening programs (CYTOBASE, Ontario Breast Screening Program, Ontario Health Insurance Plan), and neighbourhood income quintiles (Census). Direct linkage was first attempted using Ontario Health Insurance Plan (OHIP) numbers. Indirect links using names and birthdates were subsequently attempted for individuals for whom direct linkage had been unsuccessful. We were successfully able to link 76% (*N* = 259) of Inuit adults to the ICES database. A significant portion of the unlinked participants had health cards from other provinces/territories, which prevented linkage to ICES data holdings. For comparison purposes, we also drew upon ICES holdings to generate estimates of preventive health screening rates, emergency room usage, and hospitalization rates for the general populations of Ottawa and Ontario.

### Statistical analysis

Adjusted population-based estimates and confidence intervals were calculated using the RDS-I enhanced data smoothing estimator in the custom RDSAT software (version 7.1) (Volz et al. 2012). The RDS methodology anticipates that personal networks are not randomly distributed, and therefore adjusts for small to moderate levels of network clustering (people having ties to others like them), in the form of post-sampling weights.

To calculate the size of the Inuit population in Ottawa, we used a multiplier method (Johnston et al. 2013). This calculation drew on the proportions of our sample that participated, did not participate, or did not know if they participated in the 2006 Census and the 2006 Census estimate of the Ottawa Inuit population of 605 persons. We assumed that only those who reported participation had indeed participated in the 2006 Census and multiplied the 2006 Census estimate proportionally to correct for Census non-participation.

## Results

The number of Inuit adults living, working, or accessing health and social services in the City of Ottawa at the time of the study was estimated to be 3361 (95% CI 2.309–4.959). Reported participation in the 2006 Canadian Census was very low (18%, 95% CI 12.2–26.2) (Table 1).

**Table 1:** Demographic and social characteristics of Inuit adults (total *N* = 341)

Characteristic	Sample percent	RDS-adjusted prevalence, % (95% CI)
Gender		
Male	38.3	43 (33.9–52.5)
Age		
18–34	36.0	39.8 (30.1–50)
35–49	37.8	37.6 (28–47.3)
50+	26.3	22.6 (15.3–30.7)
Permanent residence		
Ontario	81.1	55.6 (40.1–67.5)
Other province/territory	18.9	44.4 (32.5–59.9)
Participation in 2006 Census		
Yes	25.4	18 (12.2–26.2)
No	53.8	59.7 (49.6–68.6)
Do not know	20.8	22.2 (14.4–30)
Education		
Some high school or less	51.8	59.2 (48.3–67.2)
Completed high school	11.6	13.9 (7.9–20.5)
Some college or more	36.6	26.9 (20.8–36.5)
Personal annual income		
Less than $20,000	68.4	69.3 (60–79.5)
$20,000 or more	31.6	30.7 (20.5–40)
Wage-earning job	40.8	46.1 (36.3–56.2)
Mobility in past 5 years		
No moves	19.9	22.3 (14–29.8)
1 move	19.3	17.8 (12–26.6)
2–3 moves	32.9	34 (24.6–42.6)
4–5 moves	14.5	13.4 (7.5–19.4)
6+ moves	13.3	12.6 (7.4–19.9)
Overcrowding		
≤ 1 person per room	86.4	80.1 (70.3–88.7)
Crowded (> 1 person per room)	13.6	19.9 (11.2–29.9)
Food insecurity		
Times when the food for the household did not last and there was no money to buy more	57.1	55 (46–64.9)

*CI* confidence interval, *RDS* respondent-driven sampling

With respect to the social determinants of health, 69.3% (95% CI 60.0–79.5) of the Inuit population in Ottawa reported an annual personal income of less than $20,000 and 53.9% (95% CI 43.8–63.8) were without a wage-earning job. One in five Inuit in Ottawa lived in overcrowded homes and 55% (95% CI 46.0–64.9) reported food insecurity (Table 1). Stratified analyses found that Inuit who were permanent residents of Ottawa at the time of the study were less likely to report food insecurity compared to Inuit who were temporary residents (35.2%; 95% CI 15.9–48.0 compared to 64.8%; 95% CI 51.5–84.3 respectively), and that Inuit who had a valid OHIP number at the time of the study were more likely to live in non-crowded homes compared to Inuit who did not have a valid OHIP number (90.0%; 95% CI 75.2–96.1 compared to 10.0%; 95% CI 3.9–25.2 respectively). Other measures of socio-demographics did not vary by residency status or OHIP registration (data available upon request). These survey measures of socio-economic deprivation were confirmed by ICES linkage results indicating that 41.5% of Inuit adults in Ottawa lived in the lowest income quintile neighbourhoods, compared to 16% of the general Ottawa population and 20% of the Ontario population (Table 2).

**Table 2:** Gender, age, and income quartiles for Inuit, Ontario-10% and the city of Ottawa

Variable	Sample			
	Inuit of Ottawa Sample (Total *N* = 259*)	Inuit of Ottawa-RDS-adjusted	Ontario-10% (Total *N* = 1,324,241)	Ottawa (Total *N* = 966,140)
	Prevalence, %	Prevalence, % (95% CI)	Prevalence, %	Prevalence, %
Gender				
Females	59.6	51.9 (38.3–62)	50.5	51.3
Males	40.4	48.1 (38–61.7)	49.5	48.7
Income quartiles				
1-Low	43	41.5 (28.1–52.1)	19.6	16.1
2	32.8	33 (24.2–48.7)	19.6	17.3
3	12.1	12.4 (4–16.2)	19.8	18.3
4	7.3	11.6 (5.1–23.1)	20.6	23.6
5-High	1.3	1.5 (0–4)	20.1	24.8

*CI* confidence interval, *RDS* respondent-driven sampling

*Total *N* reflects number of participants linked to ICES database (*N* = 259)

With respect to health status measures, the most common chronic diseases that had been diagnosed by a health care provider among Inuit adults in Ottawa included allergies (30%; 95% CI 21.4–38.3), hypertension (25%; 95% CI 18.1–33.9), chronic obstructive pulmonary disease (COPD) (6.7%; 95% CI 3.1–10.6), and cancer (6.8%; 95% CI 2.7–11.9); 41% (95% CI 33.7–51.4) had experienced severe pain and 18% (95% CI 12.2–25.7) had been injured over the past 12 months. Upper respiratory tract infection (URTI) was common, with just under 60% (95% CI 51.3–69.6) of adults reporting URTI in the past 12 months (Table 3).

**Table 3:** Prevalence of self-reported chronic health conditions and other comorbidities among Inuit adults (total *N* = 341)

Health condition	Sample percent	RDS-adjusted prevalence, % (95% CI)
Allergies	30.0	29.5 (21.4–38.3)
Arthritis	16.7	16.7 (10.9–24.5)
Bronchitis, emphysema, or COPD	7.7	6.7 (3.1–10.6)
Cancer	7.2	6.8 (2.7–11.9)
Diabetes	3.0	2.6 (0.4–8)
Heart disease	7.7	4.7 (2.1–8)
High blood pressure	24.7	25.3 (18.1–33.9)
Injury in past 12 months	23.5	18.3 (12.2–25.7)
Upper respiratory tract infection	65.0	58.8 (51.3–69.6)
Lower respiratory tract infection	8.6	6.6 (3–12.5)
Severe pain in the past 12 months	41.6	41.9 (33.7–51.4)

*CI* confidence interval, *RDS* respondent-driven sampling, *COPD* chronic obstructive pulmonary disease

In terms of access to health care, just under half (43%) of Inuit in Ottawa rated their level of access as fair or poor. Cross-cultural communication barriers, including trouble understanding what the health care provider was saying and discomfort with the health care provider because he/she was not culturally understanding of Inuit, were experienced by one in five Inuit adults in Ottawa. Additional barriers included waiting lists too long (51%), difficulty accessing traditional Inuit medicine (33%), needed follow-up/reminder call (30%), doctor not available after 5:00 p.m. and on weekends (29%), and doctor not available during business hours (28%).

**Fig. 1:**
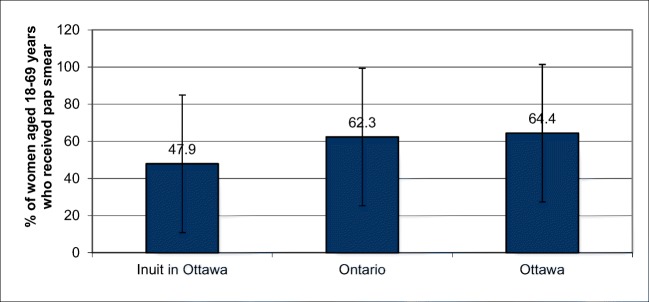
Percentage of women between 18 and 69 years who had a pap smear in the previous 3 years among Inuit of Ottawa, Province of Ontario and City of Ottawa populations

Less than half of Inuit adults in Ottawa (43%; 95% CI 33.9–52.0) reported receiving a full health review/check up with a doctor, nurse, or complementary health practitioner in the 12 months prior to the survey. Men were particularly unlikely to have accessed routine health review/check-up, with only 28% (95% CI 17.1–41.2) having done so in the past 12 months. ICES linkage data additionally suggest that Inuit women in Ottawa were less likely to have had a Pap smear than the general populations of women in Ottawa and Ontario (Fig. 1).

**Fig. 2:**
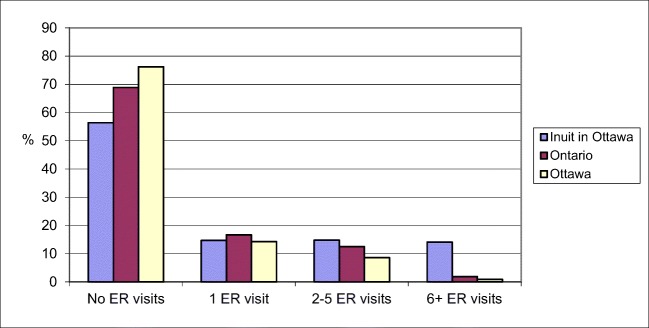
None, 1, 2–5, and 6+ emergency room visits in the previous 2 years

Rates of emergency room access were elevated for Inuit in Ottawa compared to the general Ottawa and Ontario populations overall and for both acute and non-acute conditions (Fig. 2). This higher rate of ER use for Inuit is particularly striking for persons reporting multiple (6+) ER visits. Despite comparably higher emergency room use, over 99% (95% CI 98.8–100) of the adult Ottawa Inuit population had not been hospitalized in Ontario in the past 5 years compared to 81.2% of the Ontario population and 86.0% of the general Ottawa population.

## Discussion

Our study documents a striking burden of socio-economic deprivation, chronic disease, and health care access problems for an urban Inuit population in southern Canada. Inuit community members in Ottawa have been well aware of these challenges for decades based on their lived experiences. Rates of poverty, unemployment, and food insecurity were markedly high as was relative community-level socio-economic deprivation by neighbourhood income quintile. The disproportionate burden of chronic disease included elevated rates of allergies, hypertension, and COPD. Close to half of the Inuit living in Ottawa rated their access to health care as fair or poor, and multiple barriers to health care access were identified. Finally, rates of emergency room access were elevated, and rates of hospitalization were lower for adult Inuit compared to the general Ottawa populations.

Our estimate of the number of Inuit living, working, or accessing health and social services in the City of Ottawa at the time of the study (3361; 95% CI 2.309–4.959) is more than four times that of the 2006 Canadian Census (*N* = 605). There are two key explanations for this difference. First, we found that the large majority of Inuit in Ottawa had not completed the 2006 census. This means the 2006 census significantly undercounted the number of resident Inuit in Ottawa. Second, in keeping with the needs assessment framework of our study, we purposely included Inuit who lived within the Ottawa region, but outside of Ottawa city limits who were using health and social services in the City of Ottawa.

Poverty, unemployment, food insecurity, and health care access issues have been well documented for Inuit living in northern communities (Tait 2008; Peters 2012; Inuit Tapiriit Kanatami 2007). Socio-economic deprivation has been linked to the social and economic disruption of colonization (Peters 2012; Inuit Tapiriit Kanatami 2007). For urban Inuit, there are additional complexities linked to urban transition. Health care access issues for Inuit are commonly linked to geography (Patrick and Tomiak 2008). Our study documents substantive barriers in access to culturally relevant health services despite the high density of health providers and services in Ottawa. To address this health inequity, TI has asked policy makers to work across jurisdictions to ensure that preventive, primary care, specialist, emergency room, and hospital services are accessible, culturally safe, and better matched to community needs. TI is also recommending Inuit-specific cultural safety training programs for health and social service providers working with Inuit and access for all Inuit to trained Inuit cultural interpreters (Smylie et al. 2012).

Our non-age adjusted prevalence estimates for hypertension (25%; 95% CI 18.1–33.9), COPD (6.7%; 95% CI 3.1–10.6), and cancer (6.8%; 95% CI 2.7–11.9) were elevated for Inuit in Ottawa compared to rates for the general City of Ottawa population and Canada respectively. According to the 2009 Canadian Community Health Survey, the prevalence of hypertension among adults in Ottawa was 16.0% (Statistics Canada 2009). The cancer prevalence rate in Canada in 2009 was 2.4% (Canadian Cancer Society 2018). This disproportionate burden of chronic disease is particularly disconcerting given that the Inuit population in Ottawa is much younger than the general Ottawa population and given the likelihood of under-diagnosis as a result of the access to care issues that existed at the time of our study. This burden of illness combined with the documented challenges in accessing health care services, including lower rates of routine health review/check-ups, long waiting lists, and challenges accessing physician care after hours, may be linked to the higher rates of emergency room usage. In response to these findings, TI is recommending further study to better understand and enhance the health service use pathways of Inuit accessing emergency departments in Ottawa (Smylie et al. 2012). Further investigation is also required to better understand why rates of hospitalization were lower rather than higher in relation to comparison groups, given the higher burden of illness and higher rates of ER use.

There were a few limitations of the study. First, RDS cannot guarantee a random sample of network members (Heckathorn et al. 2002); therefore, estimates are unbiased to the degree that the assumptions of the RDS estimator are met. Assumptions regarding recruiter-recruit relationships, minimum recruitment chain length, sampling with replacement, and network segregation were met in our study (Smylie et al. 2012). Additionally, RDS is a relatively new sampling method, and there is an active literature on estimation using RDS data (Heckathorn et al. 2002; Gile and Handcock 2010). There is also debate in the literature regarding the actual design effects in RDS surveys (Wejnert et al. 2012; Goel and Salganik 2010). Existing multivariable regression analyses using RDS samples have not appropriately addressed the correlation between observations and the unequal sampling probabilities inherent in RDS; therefore, we have focused on reporting prevalence estimates for which methods are better established. The majority of our results are based on self-report from a survey conducted over a 5-month period in 2010. The findings are therefore subject to self-reporting bias and temporal variation. For example, our RDS (versus sample percent) estimates of permanent versus transient residency for Inuit in Ottawa may have been impacted by an increase in the number of transient residents arriving in Ottawa at the end of the survey period which occurred in the fall season. Finally, we note there is a range of uncertainty in our estimates, with some CIs indicating less than optimal precision. Both the variance in CI width and suboptimal precision of some estimates reflect RDS methods in which weights are different for each variable, and for a given sample size, there is more variance than there would be for a random sample.

This study also has multiple strengths, including the comprehensive and ongoing partnership with TI throughout the study design, implementation, and dissemination, which optimized community participation and uptake of study results. The study results address important and persistent gaps in urban Inuit health information and clearly demonstrate that there is a larger population requiring services than indicated by census counts.

Inuit adults living in Ottawa are experiencing striking socio-economic adversity and a significant burden of chronic disease. Health services at the time of the study were poorly matched to needs, as indicated by multiple barriers in access to health care and elevated rates of emergency room use. The findings of our study were applied to the development of the new Akausivik Inuit Family Health Team (AIFHT), which currently provides medical care to Inuit in Ottawa.

## Footnotes

1 Inuit Nunangat is the homeland of Inuit of Canada. It includes the communities located in the four Inuit regions: Nunatsiavut (Northern coastal Labrador), Nunavik (Northern Quebec), the territory of Nunavut and the Inuvialuit region of the Northwest Territories. These regions collectively encompass the area traditionally occupied by Inuit in Canada.
